# Degradation of Typical PPCPs During Anaerobic Digestion and in Soil

**DOI:** 10.3390/toxics13090780

**Published:** 2025-09-15

**Authors:** Min Guo, Linyue Xu, Liguo Guo, Jie Hu, Ru Liu

**Affiliations:** Ministry of Ecology and Environment, Nanjing Institute of Environmental Science, Nanjing 210042, China; guomin@nies.org (M.G.); xulinyue@nies.org (L.X.); guoliguo@nies.org (L.G.); hujie@nies.org (J.H.)

**Keywords:** emerging contaminants, fate, influencing factors, sludge land use, risk assessment

## Abstract

Degradation is a key natural attenuation mechanism governing the fate of PPCPs during anaerobic digestion (AD) and subsequent soil exposure. Nevertheless, the combined impact of this sequential treatment (AD followed by land application) remains poorly understood. This study evaluated the degradation characteristics of nine PPCPs during mesophilic AD in three distinct soil types. The concentration changes in the nine PPCPs were monitored after 0, 5, 10, 15, 20, 25, and 30 days of anaerobic incubation at 38 °C, as well as after 0, 2, 5, 8, 10, 12, 15, 20, and 30 days of dark incubation at 25 °C with humidity at 75% in three soils. AD effectively removed sulfamethoxydiazine, ciprofloxacin, and oxytetracycline (>80%). The removal efficiencies for carbamazepine, progesterone, triclosan, naproxen, and megestrol acetate were relatively poor, with the removal rates ranging from 50% to 80%, while gemfibrozil exhibited minimal degradation (<50%). The degradation behavior of nine PPCPs fits well with first-order kinetic equations. Calculated half-lives (days) in the three soils were as follows: sulfamethoxydiazine (20.39 to 23.10), carbamazepine (36.48 to 77.02), megestrol acetate (11.18 to 20.39), progesterone (6.08 to 23.90), ciprofloxacin (11.75 to 63.01), oxytetracycline (13.08 to 30.14), naproxen (7.79 to 40.77), gemfibrozil (8.45 to 30.14), and triclosan (14.75 to 46.21). The corresponding R^2^ values ranged from 0.8882 to 0.9320 for sulfamethoxydiazine, 0.8579 to 0.9248 for carbamazepine, 0.8745 to 0.9658 for megestrol acetate, 0.9026 to 0.9560 for progesterone, 0.8147 to 0.9571 for ciprofloxacin, 0.8136 to 0.9063 for oxytetracycline, 0.8961 to 0.9156 for naproxen, 0.8802 to 0.9497 for gemfibrozil, and 0.9099 to 0.9457 for triclosan. Soil physicochemical properties significantly influenced PPCP degradation rates. Gemfibrozil warrants immediate attention due to its poor degradation; the five PPCPs presenting moderate concern—namely carbamazepine, ciprofloxacin, oxytetracycline, naproxen, and triclosan—require further risk assessment, while sulfamethoxydiazine, megestrol acetate, and progesterone pose low persistence risk according to current evidence.

## 1. Introduction

Pharmaceuticals and personal care products (PPCPs) are typical emerging organic contaminants originating from anthropogenic activities, and when unavoidably discharged into the environment, they can pose risks to ecological health [1]. PPCPs comprise a broad class of chemical compounds with diverse chemical properties that range from medications to cleaning agents, including prescription and nonprescription human drugs, illegal drugs, veterinary drugs, sunscreen, soaps, moisturizers, lipsticks, fragrances, insect repellents, and shampoo, which contain biologically active compounds and their metabolites [2]. Hundreds of tons of pharmaceutical products are manufactured and consumed worldwide. This number is continuously rising owing to the increasing population, as well as new drug and chemical discoveries and their production [3]. Hundreds of different medicines are utilized in the treatment of humans and animals, as well as in aquaculture and agriculture [4]. Many PPCPs have been detected in surface water, wastewater, sediment, and soil in a concentration range of ng/L to μg/L worldwide [5,6,7]. Individual PPCPs may not become persistent, but because these PPCPs are continuously deposited in the environment, they become “pseudo-persistent” [8]. Long-term exposure to environmental concentrations of these active compounds may affect wildlife’s wellbeing and cause multiple ecotoxicological effects, for instance, endocrine disruption, neurotoxicity, carcinogenicity, and fetal development [9,10], due to their persistence and bioaccumulation [11].

PPCPs enter the environment through multiple routes. Many PPCPs are discharged directly into the environment or released from sources such as households or hospitals, where they enter the municipal waste stream and are partially degraded in wastewater treatment facilities (WWTFs). The unclear degradation kinetics of PPCPs in activated sludge limits the efficiency of waste management [12,13]. Release of PPCPs through the discharge of wastewater effluent causes widespread environmental concern. Furthermore, effluents from WWTFs released into rivers and streams are used in agriculture for irrigation. Sewage sludge from WWTFs is often applied to agricultural fields. Residues of PPCPs and their metabolites are frequently detected in WWTF effluents and sludge [14,15]. However, there is still a lack of comprehensive understanding of the degradation behaviors of PPCPs in the environment after discharge.

Under the one health concept, understanding the degradation kinetics of multiple environmental media is important for risk assessment, waste management, and treatment. In this study, nine PPCPs with a range of physicochemical properties were selected to study degradation kinetics in anaerobic sludge and three typical soils in China because of their high detection rates in the environment [16]. Degradation characteristics of pollutants are beneficial in supporting precise ecological risk assessment and remediation efforts. Understanding the degradation behavior of these pollutants is crucial for assessing their potential impacts on ecosystems. For instance, environmental risk assessments based on degradation data can identify pollutants with high environmental risks and provide a scientific basis for their management. Furthermore, the environmental risk assessment and management of pollutants are also important areas in current research and policy-making, requiring interdisciplinary collaboration and the application of innovative technologies.

## 2. Materials and Methods

In order to investigate the fate of representative PPCPs throughout anaerobic digestion and soil exposure, we performed parallel simulation experiments with nine model compounds. The specific experimental procedures are outlined below.

### 2.1. Standards and Chemicals

The selected PPCPs are micropollutants of different therapeutic classes and physicochemical properties (log Kow ranging from −0.9 to 4.77, as detailed in Table S1). They are frequently found in municipal wastewater, whose main route of contamination is through human usage. These chemicals serve as representative examples of their respective classes, namely, the antibiotics sulfamethoxydiazine, oxytetracycline, and ciprofloxacin; the estrogens megestrol acetate and progesterone; the anticonvulsant carbamazepine; the lipid regulator gemfibrozil; the non-steroidal anti-inflammatory drug naproxen; and the disinfectant triclosan.

All analytical standards used for analysis were of high purity grade; details of the standards are provided in Table S1.

### 2.2. Anaerobic Digestion of Compounds in Sludge

**Figure 1:**
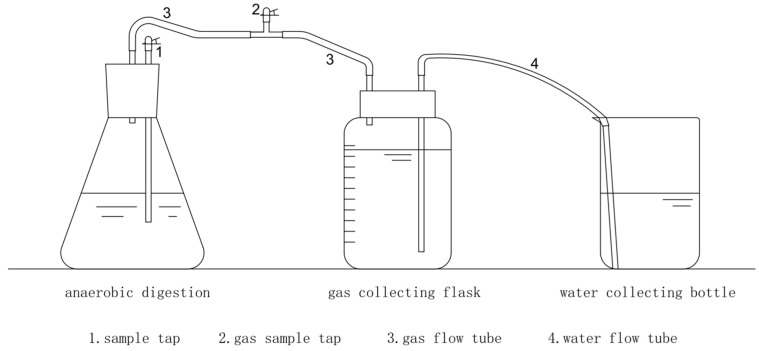
Schematic diagram of anaerobic digestion test device.

An aliquot of 240 g microbial inoculum and 38.29 g concentrated sludge was mixed in 483.4 g water in a 1 L flask to set up a reactor of anaerobic digestion with a final total solid (TS) content of 8% (Figure 1). The inoculum was incubated at 38 °C until the methane content reached 60%. The concentrated sludge was obtained from a Nanjing sewage treatment plant. The microbial inoculum was sampled from the biogas reactor at the Institute of Bioenergy of Nanjing University of Technology. The TS and volatile solid (VS) contents of the inoculum were 7.60 ± 0.02% and 4.60 ± 0.03%, respectively. The initial pH in each reactor was adjusted to 7 with 1.0 mol/L HCl or NaOH. After domestication, PPCPs were added to the system at a final concentration of 10 mg/kg. The compounds were first dissolved in acetone and then introduced into the reactor and shaken to ensure homogeneous distribution. Nitrogen was purged into the reactor to displace oxygen and ensure strict anaerobic conditions; the reactor was then incubated at 38 ± 1 °C for anaerobic digestion. The anaerobic digestion bottle was manually shaken twice a day for 1 min each time. The entire anaerobic digestion experiment was performed for 30 d. Gas production was measured daily, and sludge was sampled every 5 days to measure the concentration of the compound. All experiments were conducted in triplicate.

### 2.3. Degradation of Compounds in Soil

Soil degradation test was conducted in accordance with GB/T 31270.1-2014 [17]. Red soil, black soil, and meadow soil were selected to compare the degradational pattern of the compounds. The physicochemical properties of the soils are presented in Table 1. Soil was air-dried, pulverized, and sieved through 20-mesh sieves before use. An aliquot of 20 g of soil was added to one 150 mL triangular flask, and the moisture was adjusted to 40% of the saturated water-holding capacity of the soil. The degradation system was incubated in the dark for 2 weeks at 25 °C. After acclimation, 0.2 mL of 100 mg·L^−1^ compound was added to the system and mixed thoroughly. The solvent evaporated completely in a fume hood. The moisture was adjusted to 60% of the saturated water-holding capacity of the soil. The system was sealed with breathable silica gel stoppers and incubated at 25 °C with humidity at 75%. Samples were taken regularly to determine the residual amount of the test substance in the soils. All experiments were conducted in six replicates.

### 2.4. Instrumental Analysis of Residual Compounds

#### 2.4.1. Extraction of Dissolved Compounds in Supernatant Fraction of Sludge

The supernatant was obtained by centrifugation at 10,000 r/min for 10 min and loaded onto the activated Oasis HLB column at a rate of 5 mL/min. The column was washed with 10 mL ultrapure water and dried under vacuum for 30 min; then, the column was eluted with 10 mL of elution buffer (methanol–acetonitrile–formic acid = 20:75:5, *v*/*v*/*v*), and 1 mL of eluent was filtered through a 0.22 μm membrane for instrumental analysis. The Oasis column was activated with 5 mL of methanol and 10 mL of pure water at pH 3.5 before use.

#### 2.4.2. Extraction of Extractable Adsorbed Compounds in Solid Fraction of Sludge

The solid fraction of sludge was harvested after centrifugation at 10,000 r/min for 10 min, freeze-dried, ground, and sieved through a 20-mesh sieve. The diatomaceous earth was treated with a 0.1 M EDTA solution and dried under vacuum. In total, 2 g of the prepared sludge was mixed with 8 g of EDTA-treated diatomaceous earth and transferred into an ASE300-accelerated solvent extraction cell with a glass fiber filter membrane to prevent the powder from blocking the pipeline. The sample was extracted with 1% acetonitrile acetate (*v*/*v*) for 10 min at a pressure of 1500 psi and a temperature of 80 °C. The extract was evaporated to 2~3 mL at 40 °C and purified using 100 mg of sorbent C_18_ and 150 mg of N-Isopropylethylenediamine. Then, 1 mL of eluent was cleaned up by filtration through a 0.22 μm membrane.

#### 2.4.3. Extraction of Residual Compounds in Soil

In total, 20 g of soil was extracted three times using 20 mL EDTA-McIlvaine buffer and 20 mL of acetonitrile, sonicated for 30min, and centrifuged. The supernatant was combined, and its volume was adjusted to 500 mL with ultrapure water. The extracts were purified following the method for the solid fraction of sludge.

#### 2.4.4. Instrumental Conditions

Instrumental analysis was carried out using an Orbitrap QE Focus liquid mass spectrometer (Thermo Fisher Scientific, Waltham, MA, USA) with an electrospray interface (ESI). Acquisition of 10 μL of samples was performed in both positive (PI) and negative (NI) ionization modes. Chromatographic separation for both PI and NI was performed using an InfinityLab Proshell 120 EC-C_18_ reversed-phase column (2.1 mm × 150 mm × 2.7 um). Gradient for PI used 0.2% formic acid solution (*v*/*v*) as the aqueous phase (A) and acetonitrile as the organic phase (D), while NI used 0.05% ammonia (*v*/*v*) (A) and acetonitrile (D). The detailed gradient elution procedure is listed in Table S2. Full scan mode was applied for both PI and NI. The ion source was HESI-II, with a spray voltage of 3500 V and gasification temperature of 350 °C. The pressures of the sheath and auxiliary were set as 40 and 10 arbs, respectively. The ion transfer tube temperature was set as 325 °C, with full scan mass spectra recorded over a range of 100–1050 m/z with a resolving power of 35,000. The limit of quantification and recoveries of targeted compounds ranged from 0.10 to 3.00 μg/L and 65.87 to 111.70%, respectively.

### 2.5. Statistics

All statistical analyses were conducted in R Statistical Language v 3.6.0 (R core team). Degradation kinetics was evaluated using the PestDF package (version 0.8.13) [18].

## 3. Results and Discussion

### 3.1. Anaerobic Degradation of the Targeted PPCPs in Anaerobic Sludge

**Figure 2:**
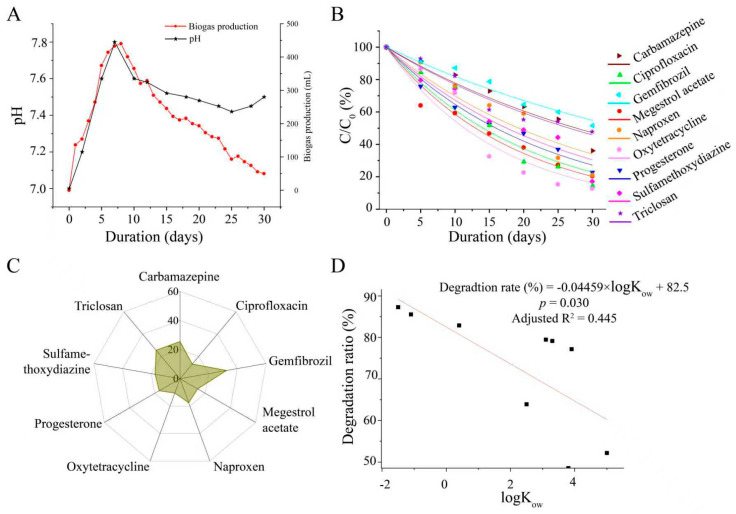
Anaerobic digestion of pharmaceutical and personal care products (PPCPs) in anaerobic sludge. (**A**) Changes in pH and single-day biogas production during digestion; (**B**) degradation curves of tested PPCPs; (**C**) T_1/2_ values in anaerobic sludge; (**D**) linear regression between logKow and degradation ratio.

The addition of compounds marginally disturbed the stability of the AD system. Sludge pH is an indicator of stability in digestion systems [19]. The pH of the reactor elevated from 7.0 to 7.8 in one week and stabilized between 7.42 and 7.60 from day 10 to 30 after the addition of compounds (Figure 2A). The pH was a little larger than the favorable range (6.5–7.5) for microbial growth and maximum production of methane during anaerobic digestion [20]. The daily production of biogas reached a peak of 440 mL at day 8 (Figure 2A). The methane production performance of mesophilic anaerobic digestion was marginally stable because of the wide variety of methanogenic bacteria in the system and better resistance to shock loads and changes in environmental conditions [21]. Although the pH increased from 7.0 to 7.8—a shift that is recognized as significant in anaerobic digestion processes—the overall system performance demonstrated notable resilience, as evidenced by the maintained methane yield.

The degradation curves of PPCPs in the AD were similar (Figure 2B), as well as the half-life values (T_1/2_) (Figure 2C). Compared to previous studies conducted at WWTFs [22], the removal of gemfibrozil, naproxen, triclosan, and carbamazepine needed a longer time under anaerobic environments. The AD system had a smaller T_1/2_ value for gemfibrozil and naproxen than the riverine ecosystem [23]. Introducing gemfibrozil-degrading bacteria could accelerate the speed of degradation of gemfibrozil (less than 16 days) [24].

A significant negative correlation was observed between the degradation ratios of PPCPs in sludge and their logKow values (R^2^ = 0.445, Figure 2D). The difference was statistically significant (*p* = 0.03). The degradation ratios of sulfamethoxydiazine, naproxen, and triclosan were 82.9%, 79.1%, and 52.2%, similar to previous studies [25,26]. The degradation ratio of carbamazepine is 63.9%, which is much greater than the results reported by Carneiro et al. [12]. Hydrophilic PPCPs (oxytetracycline, ciprofloxacin, and sulfamethoxydiazine) exhibited higher degradation ratios, with removal rates reaching 87.3%, 85.5%, and 82.9%, respectively. This enhanced degradation can be primarily attributed to their larger partition in the liquid phase, which facilitates higher bioavailability for microbial absorption and degradation [27]. The degradation ratios of carbamazepine, triclosan, and gemfibrozil (48.6%) were less than those of the other PPCPs due to their high binding to organic matter in the sludge, which may inhibit microbial bioavailability [28].

### 3.2. Degradation of PPCPs in Three Soils

As shown in Table S3, the degradation of the nine PPCPs under aerobic conditions in different soil types generally followed first-order kinetics (R^2^ > 0.8136), consistent with the previous literature findings [29], though degradation rates varied somewhat between soils. Calculated half-lives (t_1_/_2_) in soils ranged from 20.39 to 23.10 days for sulfamethoxydiazine, 36.48 to 77.02 days for carbamazepine, 11.18 to 20.39 days for megestrol acetate, 6.08 to 23.90 days for progesterone, 11.75 to 63.01 days for ciprofloxacin, 13.08 to 30.14 days for oxytetracycline, 7.79 to 40.77 days for naproxen, 8.45 to 30.14 days for gemfibrozil, and 14.75 to 46.21 days for triclosan. Compared to the half-lives during anaerobic digestion (AD)—sulfamethoxydiazine: 17.4 d; carbamazepine: 25.5 d; megestrol acetate: 14 d; progesterone: 16.5 d; ciprofloxacin: 13.1 d; oxytetracycline: 10.5 d; naproxen: 17.5 d; gemfibrozil: 32.4 d; triclosan: 25.5 d—the t_1_/_2_ values were generally higher in soils. This difference can be attributed to variations in soil versus sludge composition and properties; aeration conditions; temperatures; and, most importantly, microbial activity levels.

**Figure 3:**
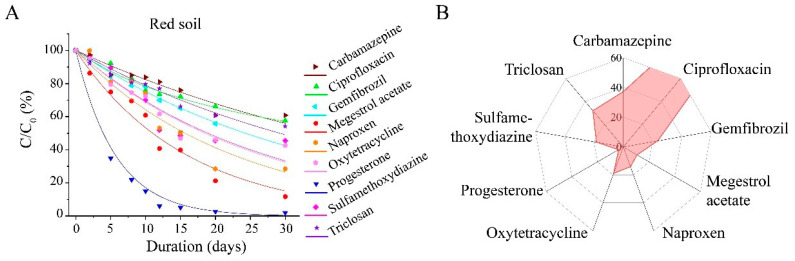
Degradation of pharmaceutical and personal care products (PPCPs) in red soils. (**A**) Degradation curves of tested PPCPs; (**B**) wind rose plot of half-lives (T_1/2_).

In the red soil of Jiangxi (Figure 3), over half of the PPCPs exhibited degradation rates exceeding 50% within 50 days. Notably, four PPCPs—sulfamethoxydiazine, megestrol acetate, progesterone, and naproxen—had half-lives ranging from 6.08 to 20.39 days. By the 10th day of the experiment, their degradation rate had reached nearly 90%, particularly for progesterone. Based on the criteria of GB/T 31270.1-2014, these four PPCPs are classified as low-persistence substances, indicating that they can be easily degraded in soil environments. The other five PPCPs, namely carbamazepine, ciprofloxacin, oxytetracycline, gemfibrozil, and triclosan, exhibit moderate degradability. Specifically, the half-lives of oxytetracycline and gemfibrozil are both 30.14 days, whereas triclosan exhibits a longer half-life of 46.21 days. Their low solubility and non-volatile nature contribute to their persistence in the environment. Additionally, these compounds are among the ten most frequently detected organic pollutants in surface water, as reported in relevant studies [30]. Ciprofloxacin and carbamazepine demonstrate relatively high persistence, each with a half-life of 63.01 days. Carbamazepine, a widely used anticonvulsant, is primarily employed for the treatment of epilepsy and neuropathic pain. Numerous studies have indicated that, due to its stable heterocyclic structure, carbamazepine is among the most persistent pharmaceuticals in both aquatic and terrestrial environments [31,32].

**Figure 4:**
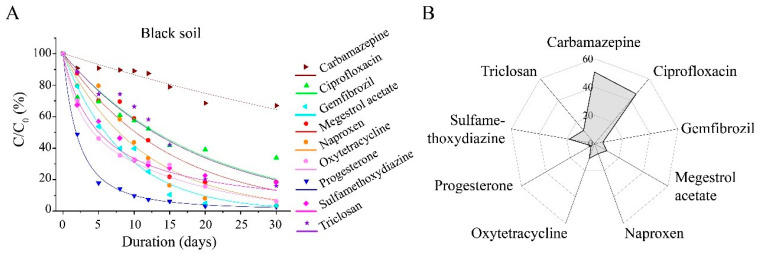
Degradation of pharmaceutical and personal care products (PPCPs) in black soils. (**A**) Degradation curves of tested PPCPs; (**B**) wind rose plot of T_1/2_ for each PPCP.

In the black soil of northeast China, the half-lives of seven PPCPs, with the exception of carbamazepine and ciprofloxacin, ranged from 7.53 to 23.10 days, indicating relatively low persistence in the black soil environment. In comparison with the half-lives in Jiangxi red soil, the half-lives of sulfamethoxydiazine, carbamazepine, megestrol acetate, and progesterone remained relatively stable at 23.10 d, 77.02 d, 12.60 d, and 7.53 d, respectively. However, the half-lives of ciprofloxacin, oxytetracycline, naproxen, gemfibrozil, and triclosan were significantly reduced by at least 50% to 36.48 d, 13.08 d, 7.79 d, 8.45 d, and 14.75 d, respectively (Figure 4).

**Figure 5:**
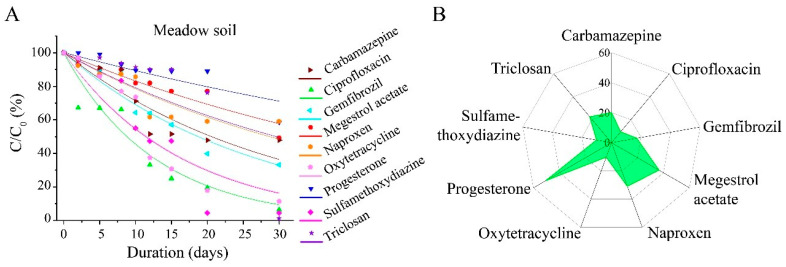
Degradation of pharmaceutical and personal care products (PPCPs) in meadow soils. (**A**) Degradation curves of tested PPCPs; (**B**), wind rose plot of T_1/2_.

As depicted in Figure 5, the half-life of sulfamethoxydiazine in meadow soil exhibits minimal variation when compared to that in red soil and black soil. Additionally, the degradation rate constant remains approximately 0.03. The half-lives of gemfibrozil and triclosan were 28.88 d and 34.66 d, respectively. These values showed little deviation from those observed in Jiangxi red soil. However, when compared to the northeastern black soil, the half-lives of both gemfibrozil and triclosan were significantly reduced by at least 50%. The half-lives of megestrol acetate, progesterone, and naproxen were approximately 1-fold longer than those in the other two soils, reaching 20.39 d, 23.90 d, and 40.77 d, respectively. Meanwhile, the half-lives of carbamazepine and ciprofloxacin were 36.48 d and 11.75 d, respectively, representing a reduction of at least 50% compared to those in Jiangxi red soil and northeastern black soil. Ciprofloxacin contains the carboxyl group (pKa = 6.09) and the amino group (pKa = 8.74), rendering it an amphoteric compound. The pH value of Jiangxi red soil is relatively low, exhibiting weak acidity. Under these conditions, the carboxyl group predominantly carries a negative charge, which effectively inhibits the hydrolysis of ciprofloxacin. The half-life of oxytetracycline was 13.33 d, a value that remained relatively consistent with that observed in black soil but was approximately 50% lower than that in red soil. Both the black soil in northeast China and the meadow soil are characterized by their high organic matter content, which results in relatively minor variability in degradation rates. The organic matter content in Jiangxi red soil is relatively low, which correlates with a slower degradation rate.

### 3.3. Impact of Soil Physicochemical Properties on the Degradability of Nine PPCPs

The degradation behavior of chemicals in soil is not only closely related to the intrinsic properties of the chemicals themselves but also significantly influenced by the soil’s physicochemical properties, such as pH, organic matter content (OC%), cation exchange capacity (CEC), and clay content. The correlation analysis between the degradation rate constants of nine PPCPs in three soils and soil physicochemical properties revealed that the degradation rate constants of sulfamethoxydiazine were significantly negatively correlated with soil pH (−0.9068), OC% (−0.9574), and CEC (−0.9883). Conversely, a positive correlation was observed with clay content (0.8759). These findings suggest that the degradation of sulfamethoxydiazine in soil is a result of the combined effects of these four physicochemical properties. Since sulfamethoxydiazine contains only the basic 4-amine aromatic (pKa = 1.98) and the acidic group sulfonamide (pKa = 7.06), it was easily ionized in soil solutions and difficult to adsorb, which resulted in low adsorption in soil and negatively correlated with the clay content [33]. The degradation rate constants of carbamazepine exhibited a moderate negative correlation with the CEC and clay content of the soil. In contrast, they showed a moderate positive correlation with the soil pH. However, no significant correlation was observed between the degradation rate constants and the OC%. Both the degradation rate constants of megestrol acetate and progesterone showed strong negative correlations with soil pH (r > -0.8)., indicating a strong negative correlation between their degradation rates and soil pH. Conversely, the degradation rates of these compounds were highly positively correlated with the clay content of the soil. Consistent with the findings of Xu et al. [34], megestrol acetate and progesterone exhibited a moderate negative correlation with soil OC%, while no significant correlation was observed with CEC. Soils with higher OC% may facilitate the sorption of these progestins onto soils, which potentially limits their biotransformation [35]. The biotransformation rates of steroid hormones are often related to their structural characteristics and soil functionalities [36]. Megestrol acetate and progesterone both have ketone groups and double bonds in their structures, which may facilitate their sorption into soils via hydrophobic partitioning and π−π bonding interactions. The degradation rate constant of ciprofloxacin exhibited a high correlation with the clay content of the soil. Meanwhile, its correlation coefficients with soil pH and OC% ranged from 0.5 to 0.8, indicating moderate positive correlations with these factors. However, no significant correlation was observed between the degradation rate constant of ciprofloxacin and the CEC of the soil. The correlation coefficients between the degradation rate constant of oxytetracycline and soil pH, OC%, and CEC were all greater than 0.9. This indicates a highly significant positive correlation between the degradation rate constant of oxytetracycline and these three soil properties. Conversely, the degradation rate constant of oxytetracycline was negatively correlated with clay content. The degradation rate constant of naproxen shows a certain positive correlation with the CEC of the soil, while no significant correlation can be observed with the other three soil parameters. This suggests that the degradation of naproxen is likely driven by the abundance of microorganisms in the soil that are capable of degrading this compound. Acidic pharmaceutical compounds, such as naproxen and gemfibrozil, containing deprotonated carboxylic functional groups, usually have more complex behavior, as their fate changes with different soil conditions [37]. The correlation coefficients between the degradation rate constants of gemfibrozil and triclosan and the CEC of the soil both exceeded 0.8, indicating a strong positive correlation between their degradation rates and soil cation exchange. Consistent with the findings of Shao et al. [38], the T_1/2_ of triclosan was relatively correlated with OC% in a positive way (0.5803), which was probably attributable to an increase in OC%, which can promote the sorption of PPCPs by soil and, hence, decrease the bioavailability of PPCPs. In contrast, their degradation rates showed nonsignificant correlation with soil pH and clay content (Table S4).

### 3.4. Fate of Degradation of Nine PPCPs in Anaerobic Sludge Digestion and Land Use Scenarios

Sludge land use is a primary method for the disposal of urban sludge, both domestically and internationally. In the future, the application of sludge in landscaping, forestry, and soil improvement is expected to become the main direction for its utilization. However, the presence of organic pollutants in sludge remains one of the key limiting factors for its widespread land use. Wilson et al. developed a mathematical model to predict the potential risks of groundwater and agricultural product contamination following the application of urban sludge to soil [39,40,41]. The model’s predictions indicated that, under normal sludge application rates and when the concentrations of organic pollutants in the sludge are below the limits specified in agricultural sludge standards, the likelihood of groundwater contamination exceeding quality standards is low. However, the risk of organic pollutants entering the food chain remains high, particularly when considering the levels of organic pollutants in the sludge and the quantity of sludge applied. Therefore, appropriate treatment of sludge is essential prior to its agricultural application to enhance the safety of urban sludge for agricultural use.

After treated sludge is applied to soil, it can be further stabilized through the soil’s self-purification capacity. Additionally, the sludge improves soil structure, enhances soil fertility, and provides essential nutrients for plant growth. Lai Huajie et al. investigated the abatement behavior of benzotriazole and 5-methylbenzotriazole in soil amended with sludge through field experiments. They found that the correlation and regularity between the amount of sludge applied and the degradation rate or half-life of the target compounds were not evident. Given the complexity of the field environment, which is influenced by multiple factors, the intrinsic properties of the compounds themselves may be the dominant factor influencing their degradation rates [42].

During this study, the degradation characteristics of the target compounds in soil were utilized to simulate the degradation trends of nine PPCPs in soils following sludge agricultural application. According to GB/T 31270.1-2014, the half-life of pesticides in soil is classified as follows: if the half-life is less than 30 days, it is considered easily degradable; if the half-life is between 30 and 90 days, it is considered moderately degradable; if the half-life is between 90 and 180 days, it is considered relatively difficult to degrade; and if the half-life is greater than 180 days, it is considered difficult to degrade. The two primary degradation pathways—anaerobic digestion and degradation in soil—in sludge land-use scenarios are summarized in Table S5. Sulfamethoxydiazine, megestrol acetate, and progesterone exhibit relatively high degradability in various soils, as well as during mesophilic anaerobic digestion. Based on their stability, these three PPCPs are unlikely to pose significant persistent pollution risks in sludge digestion and land use scenarios. In contrast, oxytetracycline, ciprofloxacin, naproxen, carbamazepine, and triclosan exhibit limited degradability during mesophilic anaerobic digestion but are moderately degradable in different soils. Given their potential persistence, further studies are needed to assess the pollution risks associated with their application in sludge land use scenarios. Gemfibrozil tends to concentrate in the solid phase during mesophilic anaerobic digestion, resulting in lower degradation rates. Although this emerging contaminant is easily to moderately degradable in various soils, its accumulation potential warrants additional research to ensure the safety of sludge agricultural use.

## 4. Environmental Implications and Limitations

The degradation ratio was primarily influenced by the chemical structure of the compounds. Specifically, aromatic compounds containing hydroxyl groups and alkylphenol compounds exhibited higher degradability. However, the differences in degradation ratios observed across studies may be attributed to the diverse structure and function of microbial communities. Therefore, it would be beneficial to characterize the microbial community within the sludge to better align and compare the conclusions drawn from different studies.

## 5. Conclusions

The degradation rates of nine PPCPs in the AD system exhibited distinct patterns. Sulfamethoxydiazine, ciprofloxacin, and oxytetracycline showed a degradation rate exceeding 80%, indicating that they are easily degradable. Carbamazepine, progesterone, triclosan, naproxen, and megestrol acetate had degradation rates ranging from 50% to 80%, suggesting that they possess moderate degradability. In contrast, the remaining PPCP, gemfibrozil, was relatively stable and exhibited minimal degradation.

The removal rate of PPCPs in the AD system is significantly correlated with their dissolution rate in the liquid phase. Specifically, the greater the migration of these compounds from the solid phase to the liquid phase, the higher the removal rate observed, and vice versa. While current research often focuses on the removal efficiency of various emerging pollutants, further investigation into the removal pathways and degradation mechanisms within sludge anaerobic digestion is essential to enhancing our understanding and improving overall treatment efficacy.

The nine PPCPs were found to undergo partial abatement in soil, with their degradation behavior in different soil types fitting well with first-order kinetic equations. These nine PPCPs covered a wide spectrum of chemical properties that defined their degradation fate. These experimental results were obtained under laboratory simulation conditions and may differ from actual degradation behavior in the field. Systematic investigation of the degradation of the relevant substances under field conditions is still needed.

Given that controlling the source of pollutants is challenging, we recommend establishing a pollutant toxicity database grounded in sludge land use research. This database should be utilized to evaluate the ecological risks associated with sludge land use. Additionally, the risk classification proposed in this paper is a preliminary outcome based on limited data; to systematically assess the risk of compounds in sludge land-use, it is essential to assess the health and ecological risks posed by pollutants introduced into the soil through sludge land use by examining their transfer characteristics along the sludge–soil–plant–human or animal pathways.

## Figures and Tables

**Table 1:** Basic physicochemical properties of the selected soils.

Soil Type	pH	Organic Matter (%)	Cation Substitution Amount (cmol(+)/kg)	Soil Particle Content ᴡ/%			Texture
				Sand Grain (2–0.05 mm)	Powder Particles (0.05–0.002 mm)	Viscous Particles (<0.002 mm)	
Red Soil	4.60	1.36	11.53	15.00	42.76	42.24	Chalk clay
Black Earth	7.28	6.54	28.87	39.04	38.80	22.16	Loamy soil
Meadow soil	7.96	6.85	22.13	11.96	73.20	14.84	Powdered sandy loam

## Data Availability

All available data can be obtained in the Supplementary Materials.

## Supplementary Materials

The following supporting information can be downloaded at: https://www.mdpi.com/article/10.3390/toxics13090780/s1, Table S1: Details of the 9 PPCPs; Table S2: Gradient elution procedure in positive and negative ion mode; Table S3: Kinetics models of PPCPs in different soils; Table S4: Linear correlation coefficients between degradation rate constants and soil properties; Table S5: Dissipation trends of 9 PPCPs in sludge land-use scenarios.
