# Supplementary material for: Degradation of Typical PPCPs During Anaerobic Digestion and in Soil
Source: Toxics. 2025 Sep 15;13(9):780. doi: 10.3390/toxics13090780 (PMC12474287; doi:10.3390/toxics13090780)
Supplement: Supplementary file 1 [file toxics-13-00780-s001.zip › toxics-3852570-supplementary.pdf]

Supplementary materials

Table S1 Details of the 9 PPCPs

Name	CAS	Category	Purity	logKow	Producers
Sulfamethoxydiazine	651-06-9	Sulfonamide antibiotics	99%	0.4	Dr. Ehrenstorfer GmbH, Germany
Carbamazepine	298-46-4	Anticonvulsants	98%	2.5	Hebei Bailingway Superfine Material Co.
Megestrol acetate	595-33-5	Estrogens	98%	3.1	Hebei Bailingway Superfine Material Co.
Progesterone	57-83-0	Estrogens	98%	3.9	Hebei Bailingway Superfine Material Co.
Oxytetracycline	79-57-2	Tetracycline antibiotics	98%	-1.5	Hebei Bailingway Superfine Material Co.
Ciprofloxacin	85721-33-1	Quinolone antibiotics	98%	-1.1	Tokyo Chemical Industry Co.
Gemfibrozil	25812-30-0	Blood lipid regulating drugs	97%	3.8	Hebei Bailingway Superfine Material Co.
Naproxen	22204-53-1	Non-steroidal anti-inflammatory drugs	98%	3.3	Hebei Bailingway Superfine Material Co.
Triclosan	3380-34-5	Disinfectants	98%	5	Hebei Bailingway Superfine Material Co.

Table S2 Gradient elution procedure in positive and negative ion mode

Mode	Time (min)	Flow rate (mL/min)	Mobile phase A (%)	Mobile phase D (%)
Positive ion	0	0.3	90	10
	2	0.3	90	10
	3	0.3	70	30
	6	0.3	50	50
	9	0.3	40	60
	15	0.3	90	100
	15.1	0.3	90	10
Negative ion	0	0.3	90	10
	2	0.3	90	10
	3	0.3	70	30
	6	0.3	50	50
	10	0.3	40	60
	10.1	0.3	90	10
	11	0.3	90	10

Table S3 Kinetics models of PPCPs in different soils

Matter	Soil type	Degradation curve equation	Correlation coefficient (R^2)	Half-life ($t_{0.5}$)/d
Sulfamethoxydiazine	Jiangxi Red Soil	C_t =0.9799$e^{-0.034t}$	0.9320	20.39
	Northeast Black Soil	C_t =0.7074$e^{-0.030t}$	0.8882	23.10
	Gansu meadow soil	C_t =1.0354$e^{-0.031t}$	0.9261	22.36
Carbamazepine	Jiangxi Red Soil	C_t =0.9651$e^{-0.011t}$	0.9248	63.01
	Northeast Black Soil	C_t =0.9588$e^{-0.009t}$	0.8579	77.02
	Gansu meadow soil	C_t =0.9768$e^{-0.019t}$	0.8890	36.48
Megestrol acetate	Jiangxi Red Soil	C_t =0.7163$e^{-0.062t}$	0.9658	11.18
	Northeast Black Soil	C_t =0.9659$e^{-0.055t}$	0.9282	12.60
	Gansu meadow soil	C_t =0.7457$e^{-0.034t}$	0.8745	20.39
Progesterone	Jiangxi Red Soil	C_t =0.5375$e^{-0.114t}$	0.9026	6.08
	Northeast Black Soil	C_t =0.5565$e^{-0.092t}$	0.9560	7.53
	Gansu meadow soil	C_t =1.0762$e^{-0.029t}$	0.9090	23.90
Ciprofloxacin	Jiangxi Red Soil	C_t =0.9078$e^{-0.011t}$	0.8160	63.01
	Northeast Black Soil	C_t =0.7464$e^{-0.019t}$	0.8147	36.48
	Gansu meadow soil	C_t =0.7359$e^{-0.059t}$	0.9571	11.75
Oxytetracycline	Jiangxi Red Soil	C_t =0.8866$e^{-0.023t}$	0.8434	30.14
	Northeast Black Soil	C_t =0.6375$e^{-0.053t}$	0.9063	13.08
	Gansu meadow soil	C_t =0.9024$e^{-0.052t}$	0.8136	13.33
Naproxen	Jiangxi Red Soil	C_t =1.0367$e^{-0.043t}$	0.9156	16.12
	Northeast Black Soil	C_t =0.8857$e^{-0.089t}$	0.9130	7.79
	Gansu meadow soil	C_t =0.8430$e^{-0.017t}$	0.8961	40.77
Gemfibrozil	Jiangxi Red Soil	C_t =1.1351$e^{-0.023t}$	0.9497	30.14
	Northeast Black Soil	C_t =0.8118$e^{-0.082t}$	0.9030	8.45
	Gansu meadow soil	C_t =0.9159$e^{-0.024t}$	0.8802	28.88
Triclosan	Jiangxi Red Soil	C_t =0.9137$e^{-0.015t}$	0.9099	46.21
	Northeast Black Soil	C_t =0.9362$e^{-0.047t}$	0.9457	14.75
	Gansu meadow soil	C_t =1.0084$e^{-0.020t}$	0.9180	34.66

Table S4 Linear correlation coefficients between degradation rate constants and soil properties

Matter	pKa	Correlation coefficient			
		pH	Organic matter content	Cation exchange capacity	clay content
Sulfamethoxydiazine	7.06/1.98	-0.9068	-0.9574	-0.9883	0.8759
Carbamazepine	13.9	0.5024	0.3744	-0.0622	-0.5600
Megestrol acetate	12.7/-3.8	-0.8035	-0.7110	-0.3380	0.8423
Progesterone	18.92/-4.8	-0.8239	-0.7351	-0.3707	0.8606
Ciprofloxacin	6.09/8.74	0.7658	0.6691	0.2802	-0.8080
Oxytetracycline	3.27/9.5	0.9755	0.9968	0.9336	-0.9581
Naproxen	4.15	-0.0335	0.1085	0.5268	0.1017
Gemfibrozil	4.42/-4.8	0.3390	0.4689	0.8041	-0.2739
Triclosan	7.9	0.4589	0.5803	0.8748	-0.3971

Table S5 Dissipation trends of 9 PPCPs in sludge land-use scenarios

Matter	Removal rate after 30 days of anaerobic digestion /%	Half-life in soils/d	Management recommendations
Sulfamethoxydiazine	82.9	20.39-23.10	Low Risk
Megestrol acetate	79.5	11.18-20.39	
Progesterone	77.2	6.08-23.90	
Oxytetracycline	87.3	13.08-30.14	It is recommended to strengthen the study on the impact of pollution application amount
Ciprofloxacin	85.5	11.75-63.01	
Naproxen	79.1	7.79-40.77	
Carbamazepine	63.9	36.48-77.02	
Triclosan	52.2	14.75-46.21	
Gemfibrozil	48.6	8.45-30.14	It is recommended to strengthen research on accumulation and sludge safety for land use
